# A bi-level dynamic emergency route planning system considering signal preemption control using CV technology

**DOI:** 10.1371/journal.pone.0323209

**Published:** 2025-05-19

**Authors:** Yulu Dai, Liang Hu, Shutong Zhou, Yanbin Liu, Aixi Yang

**Affiliations:** 1 Hangzhou Vocational and Technical College, Hangzhou, Zhejiang, China; 2 Jiangsu Provincial Department of Transportation Planning and Research Center, Nanjing, Jiangsu, China; 3 Hohai University, Nanjing, Jiangsu, China; 4 Tsinghua University, Beijing, Beijing, China; 5 Zhejiang University, Hangzhou, Zhejiang, China; Southwest Jiaotong University, CHINA

## Abstract

Emergency Vehicles (EVs) are of considerable significance in saving human lives and property damages. To promote the efficiency of emergency operation, signal preemption control could give priority to EVs heading toward the incident location. On the other hand, providing dynamic and precise route planning for EVs plays an important role in emergency rescue since traffic changes constantly. Furthermore, connected vehicle (CV) technology that incorporates advanced wireless communication technologies, offers a huge potential to promote the efficiency of EVs and maintain smooth traffic flow via collaborative optimization of routes and signals. This study presents a bi-level dynamic emergency route planning system considering signal preemption control, which builds on traffic flow combined with hierarchical bi-layer model predictive control (MPC), for more than one EV under partial CV environment. In this approach, the mobility of EVs is prioritized before decreasing the impact of EVs operation on normal traffic. In the upper layer, a road-level emergency route would be dynamically planned and updated after each time horizon, according to the network-wide traffic flow estimation under diverse CV market penetration ratios through loop detectors and Cellular-Vehicle-to-Everything (C-V2X) communication. In the lower layer, a lane-level emergency route that combined with signal preemption control would be planned to ensure the efficiency of EVs and reduce the adverse impact of signal preemption on normal traffic. In the end, a microscopic simulation environment for a real traffic network is carried out to test the effectiveness of the proposed system. The simulation results indicate that the proposed system provides reliable and practical emergency route planning and signal control services for EVs under different traffic flow conditions.

## Introduction

Once an unexpected event has occurred, an evacuation or a rescue must be carried out to provide assistance to individuals in hazardous situations, and emergency vehicles (EVs), such as fire trucks and ambulances, play an essential role in this process. Further, dynamic emergency route planning and emergency signal preemption control are two keys reducing casualties and property losses as well as even alleviating road network congestion caused by emergency rescue, which are needed to coordinate work [1–5].

Reasonable arrangement of EV route can shorten the travel time and avoid congestion, thereby the accident loss can be reduced. Regarding the minimum route distance as the target, the traditional algorithms are mainly based on historical data but this might not be always the best option for EVs in practice, since real-world traffic is constantly changing [6,7]. Among the existing studies [8–12], emergency route planning in a road network that considers the dynamic time-varying characteristics of traffic flow has been investigated. They can typically be classified into road-level route planning and lane-level route planning. Compared to road-level planning, lane-level route planning can provide more precise details that are essential for vehicles’ high precision localization and path guidance during driving [13,14]. Furthermore, many studies suggest that lane-level route planning is more efficient and applicable for vehicles possessing communication technology [7, 15–17].

In practice, in spite of dynamic route planning, EVs’ efficiency still might be disturbed by other formal vehicles (i.e., EVs move slowly since the speed of the preceding vehicle is low) and EVs even queue if the subjective signal is red. Hence, in order to guarantee EVs maintain their efficiency, some scholars proposed signal preemption control strategies based on given routes [3,13,18]. The conventional approach favored offering green waves for EVs to arrive at the destination as quickly as possible [19,20]. The wireless sensor network or radio frequency identification detects the emergency vehicles on the roadside intersection, and the traffic lights individually tune its preemption phase. In such kinds of preemption models, the traffic lights adjust their phase after the arrival of emergency vehicles. Nevertheless, emergency signal preemption control usually inevitably interrupts the normal traffic signal timing operations which could even bring superabundant delay to normal traffic [21]. For instance, an existing study [22] shows the advantages of the EVs preemption model at six locations of New York, whereas it demonstrates the disturbances at coordinated signalized intersections caused owing to preemption. Hence, minimizing the travel time without disturbing the normal road traffic is also a major purpose for EVs. Moreover, most of the signal preemption strategies for EVs are activated based on the routes that have been planned [23–26], instead of coordinated working with dynamic route planning for EVs. More specifically, most of the emergency vehicle preemption models are designed for a single emergency vehicle scenario. However, more than one emergency vehicle may present in the same lane, which is a major issue in real-time scenarios.

Dynamic route planning and coordinated signal control can better ensure the efficiency of EVs and maintain smooth traffic flow in the road network. In light of this, our study explores the potential of introducing connected vehicle (CV) technology to deal with this problem. CV technology, which contains advanced wireless communication technologies like Cellular-Vehicle-to-Everything (C-V2X), is valuable and has been gaining attention around the world [27–28]. It has been predicted that the worldwide penetration of CV in new vehicles will increase from 10% in 2018 to 70% in 2027 [29]. Under a CV environment, data is collected via sensors installed on CVs and vehicle state information (i.e., vehicle velocity, position, etc.) are guaranteed to transmit among CVs. Moreover, communication devices equipped on CVs can obtain velocity trajectories suggested by central cloud coordinator, meanwhile, the central cloud coordinator can issue instructions through C-V2X to control signals [30]. Accordingly, CV technology can provide an opportunity to assign space-time priority to EVs via dynamic route planning and signal control [31–33].

To summarize, seldom researchers conduct signal preemption control for EVs combined with dynamic emergency route planning and concerning negative impacts on normal traffic, especially under diverse CV market penetration ratios. Moreover, it is crucial to develop such a system that combines vehicles and infrastructures to improve the efficiency of emergency rescue and reduce the impacts on road networks under partial CV environment. Therefore, the objective of this study is to design a system for EVs that:

- Providing dynamic road-level route planning and lane-level route planning for EV to enhance its efficiency.
- Control signals to provide green wave for EV and reduce the influence of EV operation on normal traffic.
- Taking advantage of CV technology under diverse CV market penetration ratios.

The remainder of the paper is organized as follows: Section II ‘an integrated system of signal preemption control lane-level dynamic emergency route planning’ provides a description of the proposed system; Section III ‘Route planning algorithms and traffic signal preemption control scheme’ presents road-level route planning and lane-level route planning algorithms as well as signal preemption control scheme; Section IV ‘A study case to test the numerical model on a real network’ assesses the proposed system by applying it to a real network; Section V ‘Discussion’ presents this study’s limitations; Section VI ‘Conclusion’ concludes this paper and briefly discusses future research directions.

### An integrated system of signal preemption control and lane-level dynamic emergency route planning

**Fig 1:**
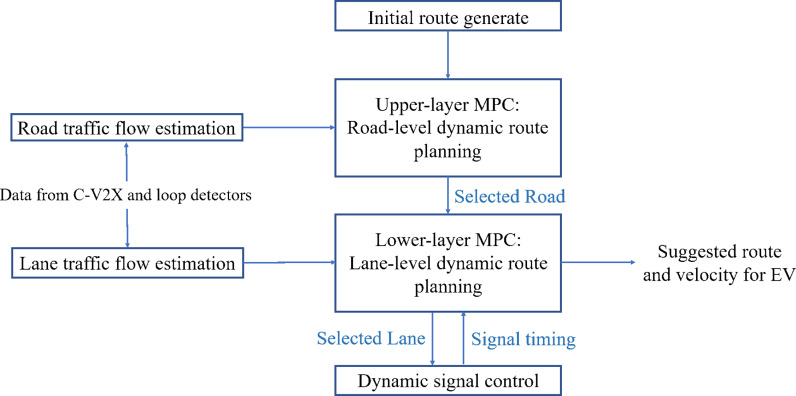
Framework of the dynamic route planning system with signal control.

In this section, the framework of the proposed integrated system is presented in Fig 1. The system is designed to operate in heterogeneous environments, leveraging existing infrastructure (e.g., loop detectors and mobile signaling data) and optionally utilizing CV technology to enhance real-time coordination between vehicles and traffic lights. Additionally, this structure is based on the assumptions that communication is reliable and there exist no communication issues such as delay and data packet loss.

The system is activated when an EV platoon makes a request. To begin with, an initial route would be generated and we refer to A* algorithm in this step. Additionally, data (i.e., numbers of CVs in the road network, etc.) is collected by C-V2X, which is leveraged to estimate traffic flow of road and lane. Then, estimation results are sent to do dynamic route planning. Due to the complexity of dynamic route planning integrating with signal preemption control, model predictive control (MPC) is introduced to assist in route planning.

**Fig 2:**
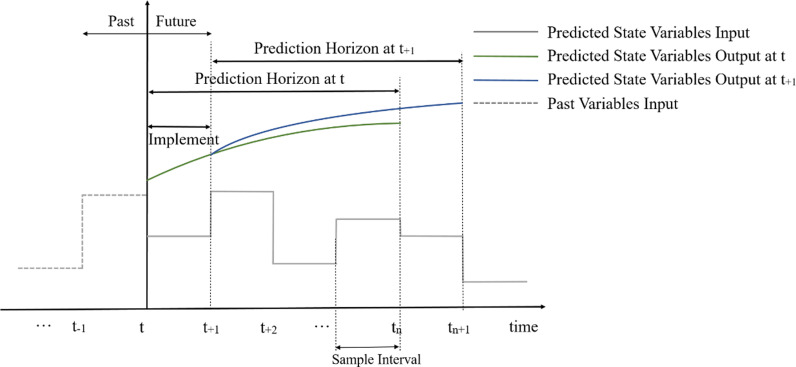
Illustration of model predictive control.

As shown in Fig 2, MPC that is an iterative, finite-horizon optimization, is appropriate for dynamic tasks. Specifically, at time t, input state variables are sampled from the past information, and then the expectation of an objective function is minimized in a certain horizon, $(\;t,t_{n}]\;$, to predict an optimal state variables sequence over the horizon. Notably, only the first predicted output state variables are implemented at time t. Then the optimization is sampled again and prediction is repeated with a shifted prediction horizon.

In this study, we design a hierarchical bi-layer MPC for dynamic route planning. The upper-layer MPC undertakes road-level dynamic route planning and provides the lower-layer MPC with selected route segments (i.e., roads and lanes). After that, the lower-lever MPC undertakes road-level dynamic route planning and offers selected lanes to traffic lights, meanwhile, traffic lights provide their signal preemption control schemes to the lower-lever MPC for its iterative prediction. In the end, the selected route that includes selected road and selected lane as well as predicted optimal velocity at time t are supplied for the EVs as the suggested route and velocity. The details of dynamic route planning and signal preemption control are elaborated in the next section.

### Route planning algorithms and signal preemption control

This section presents the process of road-level route planning and lane-level route planning as well as signal preemption control in detail. Table 1 lists the indices and parameters utilized hereafter.

**Table 1:** Indices and parameters.

Parameters	Definition
$c_{1}$	Fixed distance headway constant (km)
$c_{2}$	First variable headway constant (km^2^/h)
$c_{3}$	Second variable distance headway constant (1/h)
$C_{b}$	Signal cycle of intersection b (s)
${djam}_{p}$	Jam density on segment p (veh/km)
$d_{\_}$	Reduced delay time for EVs (s)
$D_{\_}$	Delay time for normal vehicles (s)
$g_{\min\_}$	Minimum green time (s)
$g_{\_}$	Green time (s)
ij_b	turn lane i intersection b at phase j
$k_{p}^{*},k_{d}$	Desired density of segment p (veh/km)
$k_{p}$	Real density on segment p (veh/km)
$L_{p}$	Length of segment p (km)
N	Implement horizon in MPC
${PI}_{b}$	Performance index for normal traffic after traffic control on intersection b
pa_b	EV phase pa of opposite direction on intersection b
ph_b	no-EV phase ph on intersection b
$q_{m\_p}$	Normal capacity of segment p (veh/h)
$q_{p\_out}$	Outflow of traffic on segment p (veh/h)
$q_{p\_in}$	Inflow of traffic on segment p (veh/h)
$q_{p}$	Flow of traffic on segment p (veh/h)
Q	Predicted horizon in MPC
$r_{\_}$	Red time at under signal control strategy (s)
$s_{f}$	Free-flow speed (km/h)
S	Saturation flow rate (veh/h)
${SF}_{\max}$	Maximum saturation flow of signal phase
$t_{EV\_l}$	Predicted travel time for EVs spending on the lane l (s)
${tl}_{b}$	Start-up lost time of intersection b (s)
$t_{q\_l}$	Predicted time for queue dissipation on the lane l (s)
$t_{su\_ib}$	Start-up loss time for vehicles on turn lane i intersection b
T	Sample time (s)
$v_{p}$	Speed of traffic on segment p (km/h)
$v_{p}^{*},V_{e}$	Desired speed of segment p (km/h)
V	Evaluated flow (veh/h)
${\Delta g}_{b}$	Green time for extension of intersection b (s)
$\Delta {WD}_{b}$	Weighted sum of the reduced delay time of intersection b (s)
$\Delta D_{b}$	Sum of the delay time at no-EV phase of intersection b (s)
${\Delta r}_{b}$	Compressible red time of intersection b (s)
$\lambda_{\_}$	Green ratio
α,σ, ς,l,m, $\xi ,\mu_{1},\;\mu_{2}$	Constant set according to traffic flow
τ	Reaction time for traffic flow (s)
ω	Weight for EVs
$\lambda_{\min j\_b}$	Minimum green ratio of intersection b in phase j

### Traffic flow estimation

Traffic flow estimation is vital for route planning since it can reflect whether the road capability satisfies the traffic requirement [34]. There are three important traffic parameters: speed, density, and flow. The speed and density can indicate the service quality of the road while the flow stands for how many vehicles are passing the road within a duration. The Fundamental Diagram is a macroscopic traffic model that has an equation to describe the relationship between flow, density, and speed, as


$$q_{p}=k_{p}\times v_{p}$$ (1)

In route planning, traffic flow characteristics of route segments and lanes are both fundamental. With the assistance of data from C-V2X and loop detectors, the average traffic speed of section p is described as:


$$v_{p}=v_{1}\times {(1-M)+v}_{2}\times M$$ (2)

where M is the value of CV market penetration ratio. The traffic speed $v_{1}$ is calculated by data from loop detectors installed on the midstream of all lanes, while traffic speed $v_{2}$ is calculated by data of CVs (except upstream and downstream) from C-V2X. In detail, $v_{1}$ and $v_{2}$ are calculated as:


$$v_{1}=\frac{\sum speed\;of\;vehicles\;detected\;by\;loop\;detectors}{number\;of\;vehicles\;detected\;by\;loop\;detectors}$$ (3)

$$v_{2}=\frac{\sum speed\;of\;vehicles\;detected\;by\;C-V2X}{number\;of\;vehicles\;detected\;by\;C-V2X}$$ (4)

For road-level traffic estimation, the number of vehicles stands for vehicles numbers detected of the road section, while for lane-level traffic estimation, the number of vehicles stands for vehicles numbers detected by the lane section. Based on traffic speed, we use the enhanced *Van Aerde* model [35] shown as follows to estimate the density of segments and lanes in the road network and to capture sudden congestion effects (e.g., accidents, lane closures) by integrating real-time incident data from road sensors. The revised density estimation is defined as:


$$k_{p}=\frac{1}{c_{1}+\frac{c_{2}}{S_{f}-v_{p}}+c_{3}v_{p}}+\phi \Delta k_{congestion}$$ (5)

where $\phi \Delta k_{congestion}$ is dynamically updated based on incident reports and sensor inputs, and:


$$c_{1}=Pc_{2}$$ (6)

$$P=\frac{2v_{p}-s_{f}}{{\left(S_{f}\;-\;v_{p}\right)}^{2}}$$ (7)

$$c_{2}=\frac{1}{{djam}_{p}\left(P\;+\;\frac{1}{S_{f}}\right)}$$ (8)

$$c_{3}=\frac{-c_{1}+\frac{v_{p}}{q_{m\_p}}-\frac{c_{2}}{S_{f}-v_{p}}}{s_{p}}$$ (9)

$$\begin{array}{c} \Delta k_{congestion}=\left\{ \begin{array}{cc} @ll0.5\cdot \left(d_{jam}-k_{p}\right) & if\;detect\;sudden\;congestion\\ 0 & otherwise \end{array}\right. \end{array}$$ (10)

This adjustment allows the model to rapidly adapt to abrupt traffic state changes.

### Route planning at road level

**Fig 3:**
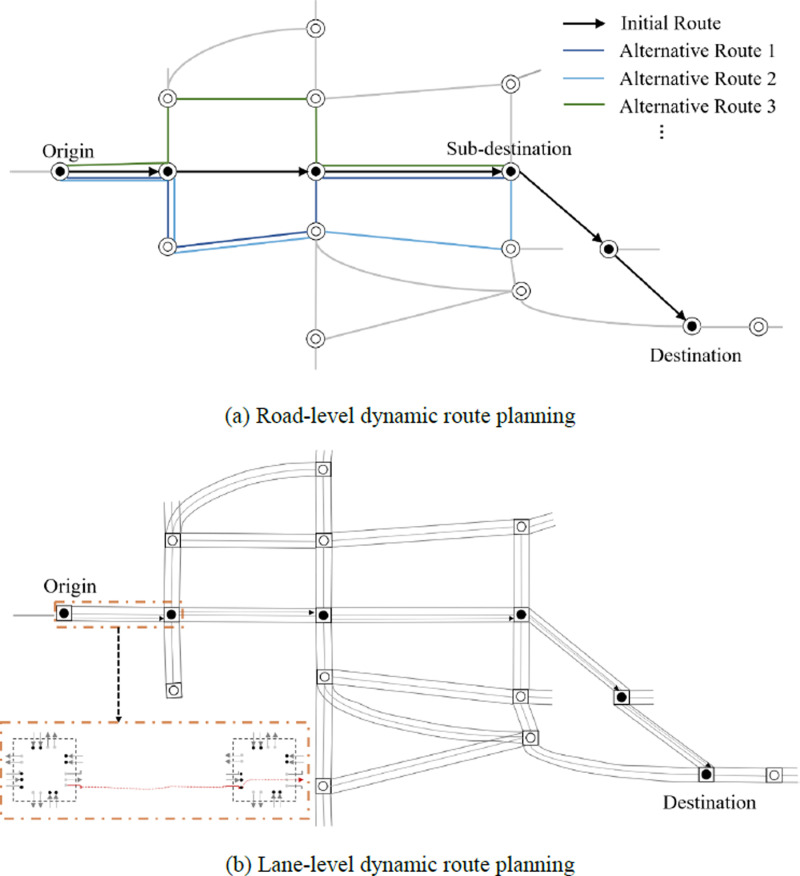
Dynamic emergency route planning based on the bi-layer MPC.

Since the state of traffic changes over time, dynamic route planning in the emergency response context is indispensable. Fig 3 shows the two steps in dynamic emergency route planning. At first, we plan route at road level, as Fig 3(a). The intersections are regarded as nodes in the road network. Specifically, according to the initial route, where paths and nodes are recorded, several following nodes are taken into consideration from the position of the EV to find the local optimal route. To simplify, in this paper, we take four nodes as an example at each sampling time for local road-level route planning. Note that, the position of the EV is denoted as origin and the fourth following node in the initial route from the origin is labeled as sub-destination. Then, all alternative routes that include less than seven nodes (maintain the same origin and the sub-destination) are provided for the EV to select. According to the traffic state estimation results of all alternative routes, the upper-layer MPC would output the travel time for the EV on each route. The route that costs the least time is selected as the suggested route segment and lane-level route planning will be executed. When the EV arrives at sub-destination, origin and sub-destination will be updated and the next stage of road-level route planning will be carried out. The above road-level dynamic route planning algorithm is summarized in Table 2.

**Table 2:** Pseudo-code for road-level dynamic route planning.

Algorithm 1 Road-level dynamic route planning
**Input:** Initial route including origin and destination, all nodes and paths in the road network as well as state of the EV
**Output:** Suggested route segment with nodes and paths
**while** nodes in the initial route exceed four from origin to destination **do**
*Set* the fourth following node from the origin in the initial route as sub-destination
**if** nodes in a route segment from the origin to sub-destination exceed six **then**
*Set* as an alternative route
*Collect* traffic data of the route
*Estimate* traffic density and speed of the route
*Predict* travel time for the EV spending on the route segment via upper-layer MPC
**end**
*Compare* the cost time in all alternative routes and select the least one as the suggested route
*Execute* lane-level route planning and signal preemption control for the EV
*Update* origin **end**

Herein, the operation process of MPC is elaborated as follows. Assume that at time n, there exist equations as:


$$\delta k_{p}(n)=k_{p}(n)-k_{p}^{*}(n)$$ (11)

$$\delta v_{p}(n)=v_{p}(n)-v_{p}^{*}(n)$$ (12)

where


$$\mathrm{\backslash [}\left[k_{p}^{*}\;(n)\;,\;v_{p}^{*}\;(n)\right]=\left[k_{d},V_{e}\left[k_{p}\;(n)\right]\right]\mathrm{\backslash ]}$$ (13)

There is a relationship between the density, flow and speed of two connected segments at time n and that at time n+1 according to traditional macroscopic traffic flow model [34,36]:


$$k_{p}\left(n\;+\;1\right)=k_{i}(n)+\frac{T}{L_{p}}\left[q_{p-1}\;(n)-q_{p}(n)+q_{p\_in}(n)-q_{p\_out}(n)\right]$$ (14)

$$q_{p}(n)=\alpha k_{p}(n)v_{p}(n)+\left(1\;-\;\alpha \right)k_{p+1}(n)v_{p+1}(n)$$ (15)

$$v_{p}\left(n\;+\;1\right)=v_{p}(n)+\frac{T}{\tau }\left\{V_{e}\left[k_{p}\;(n)\right]-v_{p}(n)\right\}-\frac{\mu (n)T}{L_{p}}\omega_{p}(n)+\frac{T}{L_{p}}\frac{k_{p-1}(n)v_{p-1}(n)}{k_{p}(n)+\xi }\left[\sqrt{v_{p-1}v_{p}(n)}\;-\;v_{p}\;(n)\right]$$ (16)

where


$$\mu (n)=\left\{ \begin{array}{c} \mu_{1}\frac{\eta }{d_{m}-k_{p+1}(n)+\sigma },k_{p+1}(n)>k_{p}(n)\\ \;\mu_{2},k_{p+1}(n)\leq k_{p}(n) \end{array}\right.\;$$ (17)

$$\omega_{p}(n)=\frac{k_{p+1}(n)-k_{p}(n)}{k_{p}(n)+\varsigma }$$ (18)

$$V_{e}\left[k_{p}\;(n)\right]=v_{f}{\left\{1\;-\;{\left[\frac{k_{p}(n)}{d_{m}}\right]}^{l}\right\}}^{m}$$ (19)

Note that, if there not exist $q_{p-1}$,$\;k_{p-1}$, $k_{p+1}$ nor $v_{p-1}$, the value of them are set as the desired value of these parameters. Hence, the relationship can be expressed in the following way:


$$\delta k_{p}\left(n\;+\;1\right)=\delta k_{p}(n)+\frac{T}{L_{p}}\left[q_{p-1}\;(n)-q_{p}(n)+q_{p\_in}(n)-q_{p\_out}(n)\right]$$ (20)

$$\begin{array}{cc} \delta v_{p}\left(n\;+\;1\right) & =\delta v_{p}(n)+\frac{T}{\tau }\left\{V_{e}\;\left(k_{d}\right)\;+\;\Gamma \;\delta \;k_{p}\;(n)\;-\;\delta \;v_{p}\;(n)\;-\;V_{e}\;\left(k_{d}\right)\right\}\\ & +\frac{T}{L_{p}}\frac{\Delta_{1}\Delta_{2}}{\delta k_{p}(n)+k_{d}+\xi }\left[\sqrt{\Delta_{2}\Delta_{3}}\;-\;\Delta_{3}\right]-\frac{\mu (n)T}{\tau L_{p}}\frac{\delta k_{p+1}(n)-\delta k_{p}(n)}{\delta k_{p}(n)+\varsigma } \end{array}$$ (21)

where


$$\Delta_{1}=\delta k_{p-1}(n)+k_{d}$$ (22)

$$\begin{array}{c} \Delta_{2}=\delta v_{p-1}(n)+V_{e}\left(k_{d}\right) \end{array}$$ (23)

$$\Delta_{3}=\delta v_{p}(n)+V_{e}(k_{d})$$ (24)

$$\Gamma =\frac{dV_{e}\left(k_{p}\;(n)\right)}{dk_{p}(n)}|k_{d}=V_{e}^{\prime }(k_{d})$$ (25)

$$\begin{array}{c} \mu (n)=\left\{ \begin{array}{c} @l\mu_{1},\frac{\eta }{d_{m}-\delta k_{p+1}(n)+\sigma },\delta k_{p+1}(n)>k_{p}(n)\\ \mu_{2},\delta k_{p+1}(n)\leq k_{p}(n) \end{array}\right.\; \end{array}$$ (26)

Using Eqs. (17) and (18), the state space representation of the traffic system can be obtained:


$$\left[\begin{array}{c} \delta k_{p}\left(n\;+\;1\right)\\ \delta v_{p}\left(n\;+\;1\right) \end{array}\right]=[\begin{array}{cc} 1 & 0\\ \frac{T\Gamma }{\tau } & 1-\frac{T}{\tau } \end{array}]\left[\begin{array}{c} \delta k_{p}(n)\\ \delta v_{p}(n) \end{array}\right]+[\begin{array}{cc} 1 & 0\\ 0 & 1 \end{array}]\left[\begin{array}{c} @lg_{p}(n)\\ f_{p}(n) \end{array}\right]$$ (27)

where


$$f_{p}(n)=\frac{T}{L_{p}}\frac{\Delta_{1}\Delta_{2}}{\delta k_{p}(n)+k_{d}+\xi }\left[\sqrt{\Delta_{2}\Delta_{3}}\;-\;\Delta_{3}\right]-\frac{\mu (n)T}{\tau L_{p}}\frac{\delta k_{p+1}(n)-\delta k_{p}(n)}{\delta k_{p}(n)+k_{0}+\varsigma }$$ (28)

$$g_{p}(n)=\frac{T}{L_{p}}\left[q_{p-1}\;(n)-q_{p}(n)+q_{p\_in}(n)-q_{p\_out}(n)\right]$$ (29)

Set $f_{p}(nand\;g_{p}(n)$ as predicted state variables outputs. Thus, Eqs. (17) and (18) can be formulated as:


$$\delta k_{p}\left(n\;+\;1\right)=\delta k_{p}(n)+f_{p}(n)$$ (30)

$$\mathrm{\backslash [}\delta v_{p}\left(n\;+\;1\right)=\frac{T\Gamma }{\tau }\delta k_{p}(n)+\left(1\;-\;\frac{T}{\tau }\right)\;\delta v_{p}(n)+g_{p}(n)\mathrm{\backslash ]}$$ (31)

Moreover, the objective function in the MPC is written as:


$$\min_{u(n),...,u\left(n\;+\;t\;-\;1\right)}J(n)$$ (32)

$$s.t.u(n)\in \left[u_{\min},\;u_{\max}\right]$$

u(n + t),u(n + t + 1),...,u(n + Q)=u(n + t − 1) (33)

where


$$J_{upper}(n)=\sum_{p=1}^{N}\sum_{q=0}^{Q}\left[{\left\|k_{p}\;(n\;+\;q\;+\;1-\;k_{p}^{*}\right\|}^{2}\;+\;\left\|v_{p}\;\left(n\;+\;q\;+\;1\right)\;-\;v_{p}^{*}\right\|\right]+\varsigma \sum_{b=1}^{M}Feedback(q_{b})$$ (34)

$$u(n)=[g_{p}(n)f_{p}(n){]}^{T}$$ (35)

where $Feedback(q_{b})$ represents lane-level queue lengths at intersection b, and ς balances route efficiency and congestion avoidance. Therefore, the problem can be solved by Quadratic Programming [37].

After the suggested route segment is provided, we plan route at lane level as Fig 3(b). In this step, we combined lane selection with signal control which guarantees the EV’s efficiency without lane changing during driving. The details of the signal control scheme and lane-level route planning follow.

### Route planning at lane level considering Signal preemption control

In normal, vehicles decelerate for lane changing which might cost more travel time. In this study, the optimal lane for the EV is suggested through the lane-level route planning algorithm. Moreover, to enhance the efficiency of the EV, a traffic light provides an applicable signal to guarantee that the EV could pass through multiple intersection without queueing and reduce deceleration performance. Although improving the efficiency of the EV is vital, the impact of EV operation on normal traffic is essential to be taken into consideration in signal preemption control. Therefore, we employ two signal control strategies combined with lane-level route planning to decrease the impact of EV operation on normal traffic as well as ensure the EV’s efficiency.

**Fig 4:**
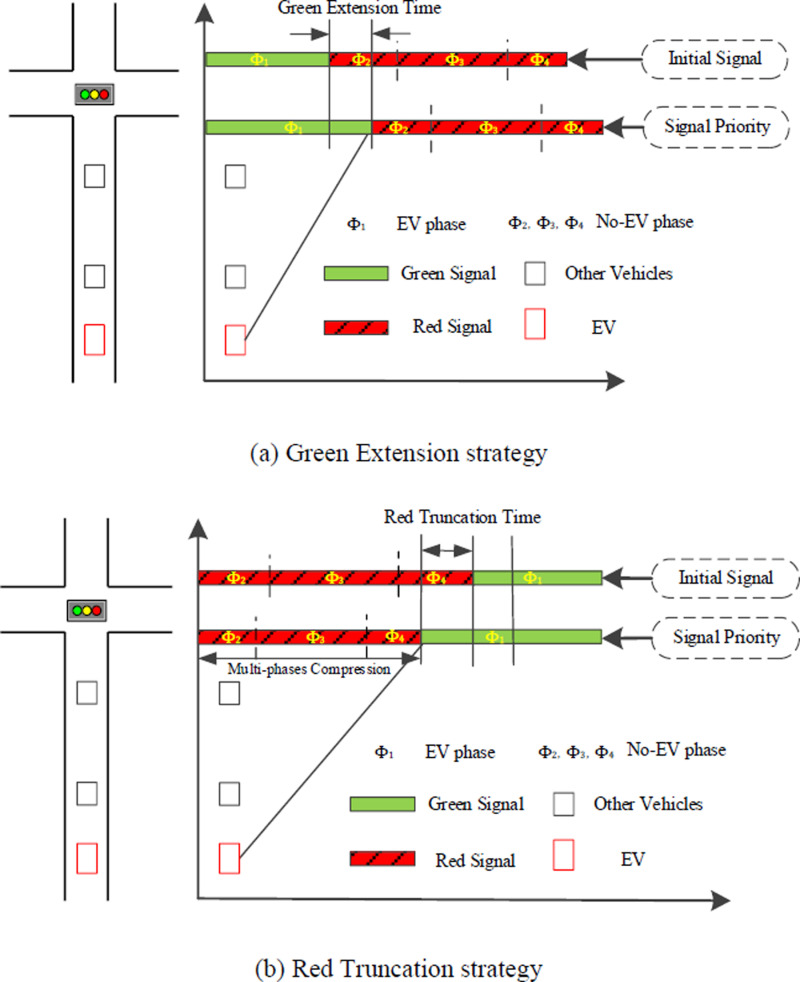
Strategies for signal control.

The process of lane-level route planning is utilized through lower-layer MPC which is similar to the upper-layer MPC except that the predicted state variables and past state variables belong to lane-level traffic flow. Further, each time after predicting arrival time through MPC, whether execute signal control and which control strategy is executed depends on the arrival phase and the value of ${PI}_{b}$ that measures the overall benefits of the subjective intersection b after signal control. If the predicted arrival phase of the EV is green, there is no need for signal control; otherwise, signal control strategies will be considered. One of the two signal control strategies leveraged in this study is Green Extension, as Fig 4 (a), while the other is Red Truncation shown as Fig 4 (b). More specifically, the Green Extension strategy is carried out if the following three requirements are met:

- The arrival time of the last EV in EVs platoon is later than the ending time of its corresponding green phase.
- After Green Extension, the saturation flow of each phase is less than ${SF}_{\max}$.
- The value of ${PI}_{b}$ is greater than zero.

In this paper, we use ${PI}_{b}$ as the performance index for normal traffic after traffic control of intersection b. Note that the value of ${PI}_{b}$ represents the weighted sum of delay for both normal vehicles and the EV at the intersection b:


$${PI}_{b}=\Delta {WD}_{b}-\Delta D_{b}$$ (36)

where $\Delta D_{b}$ is the sum of the delay time at no-EV phase of intersection b, and $\Delta {WD}_{b}$ is the weighted sum of the reduced delay time at EV phase of intersection b. Thus, when value of ${PI}_{b}$ is greater than zero, it means that the adverse influence caused by traffic control is acceptable. The calculative process for ${PI}_{b}$ is elaborated as follow:

According to the Highway Capacity Manual 2016 (HCM2016) [26], the relationship between $\lambda_{j\_b}$, $C_{b}$, and $S_{ij\_b}$ is described as:


$$\left\{ \begin{array}{c} \lambda_{j\_b}\geq g_{min\_bj}/C_{b}\\ \lambda_{j\_b}\geq V_{ij\_b}/(S_{ij\_b}\times {SF}_{\max}) \end{array}\right.\;$$ (37)

$$\lambda_{\min j\_b}=max(\frac{g_{min\_bj}}{C_{b}},\frac{V_{ij\_b}}{S_{ij\_b}\times {SF}_{\max}})$$ (38)

Then the green time for extension can be calculated as:


$${\Delta g}_{b}=C_{b}-{tl}_{b}-C_{b}\sum_{i=1}^{n}\lambda_{\min j\_b}$$ (39)

Thus, the reduced delay time for the EV under Green Extension, which is equal to the time for the EV to queue without signal control, can be written as:


$$d_{b}={\Delta g}_{b}+r_{ph\_b}+{tl}_{b}$$ (40)

**Fig 5:**
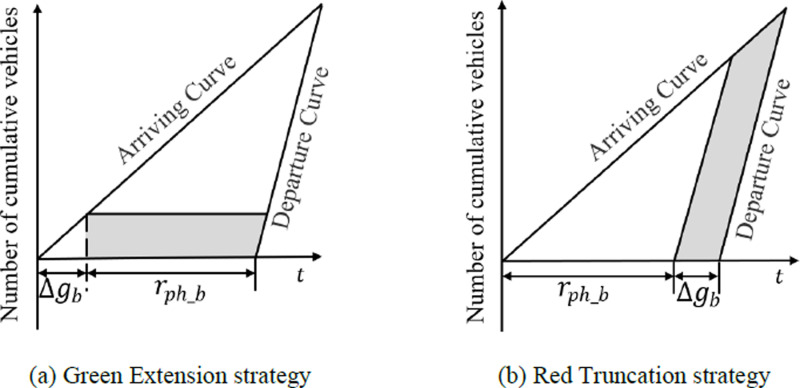
Illustration of arrival curves and departure curves under signal control.


The above equation describes the waiting time in line when the EV does not enjoy preemption. In addition, after the Green Extension strategy is adopted, some normal vehicles in this direction also get more time for passing through the intersection without waiting for the next cycle. In this case, the illustration of cumulative arrival curve and departure curve are shown as Fig 5(a). According to these curves, the reduced delay time for normal vehicles after Green Extension on turn lane i in phase j is formulated as:

$$d_{ij}=\frac{1}{2}{\Delta g}_{b}V_{ij\_b}\left({\Delta g}_{b}+2r_{ph\_b}+2{tl}_{b}+\frac{{\Delta g}_{b}V_{ij\_b}}{S_{ij\_b}}\right)$$ (41)

Provided that the number of turn lanes in intersection b is n and the number of no-EV phases in intersection b is m, then, the weighted sum of the reduced delay time of intersection b can be calculated as:


$$\mathrm{\backslash [}\Delta {WD}_{b}=d_{b}\cdot \omega +\sum_{i=1}^{n}d_{ij\_b}=\left({\Delta g}_{b}+r_{ph\_b}+{tl}_{b}\right)\omega +\sum_{i=1}^{n}\left[\frac{1}{2}{\Delta g}_{b}V_{ij\_b}\left({\Delta g}_{b}+2r_{ph\_b}+2{tl}_{b}+\frac{{\Delta g}_{b}V_{ij\_b}}{S_{ij\_b}}\right)\right]\mathrm{\backslash ]}$$ (42)

Note that the weight ω is determined by the urgency of the emergency rescue. Thus, delay time for normal vehicles on turn lane i intersection b at phase z and the sum of the delay time at no-EV phase of intersection b are obtained:


$$D_{iz\_b}=\frac{{\Delta g}_{b}S_{iz\_b}V_{iz\_b}}{S_{iz\_b}-V_{iz\_b}}\left(r_{pa\_b}+\frac{1}{2}{\Delta g}_{b}\right)$$ (43)

$$\mathrm{\backslash [}\Delta D_{b}=\sum_{z=1}^{m}\sum_{i=1}^{n}\left[\frac{{\Delta g}_{b}S_{iz\_b}V_{iz\_b}}{S_{iz\_b}-V_{iz\_b}}\left(r_{pa\_b}+\frac{1}{2}{\Delta g}_{b}\right)\right]\mathrm{\backslash ]}$$ (44)

Hence, the value of ${PI}_{b}$ under Green Extension strategy is calculated as:


$$\begin{array}{cc} {PI}_{b} & =\Delta {WD}_{b}-\Delta D_{b}=\left({\Delta g}_{b}+r_{ph\_b}+{tl}_{b}\right)\omega +\sum_{i=1}^{n}\left[\frac{1}{2}{\Delta g}_{b}V_{ij\_b}\left({\Delta g}_{b}+2r_{ph\_b}+2{tl}_{b}+\frac{{\Delta g}_{b}V_{ij\_b}}{S_{ij\_b}}\right)\right]\\ & -\sum_{z=1}^{m}\sum_{i=1}^{n}\left[\frac{{\Delta g}_{b}S_{iz\_b}V_{iz\_b}}{S_{iz\_b}-V_{iz\_b}}\left(r_{pa\_b}+\frac{1}{2}{\Delta g}_{b}\right)\right] \end{array}$$ (45)

Alternatively, the Red Truncation strategy is carried out if the following five requirements are met:

- The arrival time of the last EV in EVs platoon is earlier than the starting time of its corresponding red phase.
- All green time in no-EV phases is greater than the minimum green time $g_{min\_bj}$.
- The compressible red time is greater than zero.
- After Red Truncation, the saturation flow of each phase is less than 0.98 and the time for queue dissipation on the subjective lane $t_{q\_l}$ is less than the EV travel time $t_{EV\_l}$.
- The value of ${PI}_{b}$ is greater than zero.

In this case, the definition of ${PI}_{b}$ is same as that under Green Extension strategy. More specifically, based on Eq. (35), the compressible red time can be calculated as:


$${\Delta r}_{b}{=d}_{b}=C_{b}-{tl}_{b}-C_{b}\lambda_{\min j\_b}$$ (46)

Under Red Truncation strategy, normal vehicles in the same phase as the EV can also pass through the intersection in advance and the illustration of cumulative arrival curve and departure curve are shown as Fig 5(b). The shaded area represents the increased delay time for normal vehicles after Red Truncation on turn lane i in phase j and the weighted sum of the increased delay time $\Delta {WD}_{b}\;$are formulated as:


$$d_{ij\_b}=\frac{{\Delta r}_{b}S_{ij\_b}V_{ij\_b}}{S_{ij\_b}-V_{ij\_b}}\left(r_{ph\_b}-\frac{1}{2}{\Delta r}_{b}\right)$$ (47)

$$\Delta {WD}_{b}=\omega d_{b}+\sum_{i=1}^{n}d_{ij}=\omega {\Delta r}_{b}+\sum_{j=1}^{n}\left[\frac{{\Delta r}_{b}S_{ij\_b}V_{ij\_b}}{S_{ij\_b}-V_{ij\_b}}\left(r_{ph\_b}-\frac{1}{2}{\Delta r}_{b}\right)\right]$$ (48)

After Red Truncation, delay time for normal vehicles on turn lane i intersection b at phase z and the sum of the delay time at no-EV phase of intersection b are shown as:


$$D_{iz\_b}=\frac{{\Delta r}_{b}S_{iz\_b}V_{iz\_b}}{S_{iz\_b}-V_{iz\_b}}\left(r_{pa\_b}+\frac{1}{2}{\Delta r}_{b}\right)$$ (49)

$$\Delta D_{b}=\sum_{z=1}^{m}\sum_{i=1}^{n}\left[\frac{{\Delta r}_{b}S_{iz\_b}V_{iz\_b}}{S_{iz\_b}-V_{iz\_b}}\left(r_{pa\_b}+\frac{1}{2}{\Delta r}_{b}\right)\right]$$ (50)

Then, the value of ${PI}_{b}$ under Red Truncation strategy is calculated as:


$${PI}_{b}=\Delta {WD}_{b}-\Delta D_{b}=\omega {\Delta r}_{b}+\sum_{i=1}^{n}\left[\frac{{\Delta r}_{b}S_{ij_{b}}V_{ij_{b}}}{S_{ij_{b}}-V_{ij_{b}}}\left(r_{ph\_b}-\frac{1}{2}{\Delta r}_{b}\right)\right]-\sum_{z=1}^{m}\sum_{i=1}^{n}\left[\frac{{\Delta r}_{b}S_{iz\_b}V_{iz\_b}}{S_{iz\_b}-V_{iz\_b}}\left(r_{pa\_b}+\frac{1}{2}{\Delta r}_{b}\right)\right]$$ (51)

Additionally, based on Wang’s research [38], the predicted time $t_{q\_l}$for queue dissipation on the subjective lane l is calculated as:


$$t_{q\_l}=\frac{q_{l\_in}\times r_{ph\_b}}{S_{ij\_b}}+t_{su\_ib}$$ (52)

The lower-layer MPC not only optimizes EV travel time but also penalizes excessive queuing in general traffic lanes. The objective function includes:


$$min\;J_{lower}=\vartheta \sum_{b=1}^{N}{Queue}_{b}$$ (53)

where ${Queue}_{b}$ is the predicted queue length at intersection *b*, and ϑ balances EV priority and traffic fairness.

The above lane-level dynamic route planning algorithm with signal preemption control is summarized in Table 3.

**Table 3:** Pseudo-code for lane-level dynamic route planning considering signal preemption control.

Algorithm 2 Lane-level dynamic route planning with signal preemption control
**Input:** Suggested route segment by upper-layer MPC and initial signal timing scheme of each intersection in the route segment as well as state of all EVs in the EV platoon
**Output:** Suggested lane of each path and the signal preemption control scheme for intersections in the route segment
**while** the EV has not entered the last path in the route segment **do**
*Confirm* the direction $d_{1}$ for the EV to enter the next path
*Count* the number of lanes ${num}_{1}$ in the direction $d_{1}$
*Count* the number of lanes ${num}_{2}$, ${num}_{3}$, ⋯ ${num}_{x}$ in other directions $d_{2}\cdots$ $d_{x}$
**begin** lane choosing
**for** direction=$d_{1},d_{2}\cdots {,d}_{x}$ **do**
**for** lane=${num}_{1},{num}_{2}\cdots {,num}_{x}$ **do**
*Predict* travel time$\;t_{EV\_l}\;$for the EV spending on the lane via lower-layer MPC
*Calculate* the arrival time for the EV to enter the intersection
**if** the arrival time is included in the green time **then**
No signal control
*Stop* lane choosing
*Go* to velocity suggesting (line 34)
**else if** the green time in the current direction is greater than $g_{min\_bj}$ **then**
*Calculate* compressible red time ${\Delta r}_{b}$and ${PI}_{b}$
*Predict* time for queue dissipation on the subjective lane $t_{q\_l}$
**if** ${PI}_{b}$ and ${\Delta r}_{b}\;$are both greater than zero and $t_{q\_l}$ is less than $t_{EV\_l}$ **then**
*Do* Red Truncation strategy
**end**
*Go* to velocity suggesting (line 34)
**else**
*Calculate* the green time for extension ${\Delta g}_{b}$
**if** the saturation of the subjective lane is less than 0.98 **then**
*Calculate* ${PI}_{b}$
**if** the value of ${PI}_{b}$ is great than zero **then**
*Do* Green Extension strategy
**end**
*Go* to velocity suggesting (line 34)
**end**
**end** **end**
**end** **end**
**begin** velocity suggesting
*Predict* optimal velocity for the EV at the subjective time via lower-layer MPC
*Provide* the suggested velocity for the EV **end**
**end**
*Execute* lane-level route planning for the EV and signal preemption control scheme for the traffic signal
*Update* lanes in next path and signal timing scheme of next intersection

### A study case to test the numerical model on a real network

This section examines the effectiveness of the proposed system based on the simulation software MATLAB. We consider five EVs in the same lane in a realistic scenario which is the region with 179 intersections in Kunshan, China. Herein, based on the origin and the destination, A* algorithm is employed to generate the initial route. Moreover, the initial signal timing and lanes number of each intersection are obtained from ground truth dataset. For simplicity, the value of desired speed, the value of desired density and the value of start-up lost time as well as the value of free flow speed for vehicles respectively are the same on all lanes in this road network during simulation. Assume that all vehicles follow vehicle kinematics equation and the distribution of vehicles on the road network at the initial time is Gaussian. The specific parameters and values are presented in Table 4.

**Table 4:** Parameters and values used in experiments.

Parameter	α	σ	τ	$v_{p}^{*}$	$k_{p}^{*}$	${tl}_{b}$	ξ
Value	0.95	16	20.4 s	57 km/h	34 veh/(km*lane)	2 s	50 veh/km
Parameter	Q	N	$\mu_{2}$	ς	$d_{m}$	ω	${SF}_{\max}$
Value	10	1	6 km^2^/h	50 veh/km	110 veh/km	3	0.98
Parameter	l	m	T	$s_{f}$	M		
Value	1.86	4.05	15 s	93.1 km/h	50%		

In order to better demonstrate the validity of the proposed system, we perform numerical simulation experiments under different flow of traffic environments. The traffic flow conditions are set sequentially for 550 vehicles/(hour*lane), 1100 vehicles/(hour*lane) and 1600 vehicles/(hour*lane) to stand for low traffic case, medium traffic case and high traffic case, respectively according to the previous study [39,40]. Considering the randomness in signal time and vehicle arrival, each case is repeated multiple times and the results are averaged. Some indexes, i.e., delay time of each intersection for normal traffic and average delay time of the road network for normal traffic, in each case are demonstrated in Table V. Note, among the approaches in Table V, only A* means the EV travel on the initial route without lane suggestion and signal control; only signal preemption with A* means providing signal preemption in the initial route calculated by A* for the EV taking no account of passing through lanes for other directions at each intersection; lane-level route planning without signal control means providing lane-level route planning only according to the minimal EV’s traveling time in each road section; only signal preemption with road-level route planning means providing the EV with signal preemption under dynamic road-level route planning but dismissing passing through lanes for other directions at each intersection. Specifically, the average delay time of each intersection for normal traffic is described as:


$$Average\;delay\;time\;of\;each\;intersection=\;\frac{\sum_{b=i}^{number\;of\;intersections\;the\;EV\;passing}\Delta D_{b}}{number\;of\;intersections\;the\;EV\;passing}$$ (54)

To analyze the impact of signal preemption on general traffic, two new performance metrics: General Traffic Delay Change Rate (GTDCR) and Intersection Queue Length (IQL), are introduced in this study. The value of GTDCR can be expressed as Eq. 55 and IQL represents the maximum queue length during preemption cycles.


$$GTDCR=\frac{D_{post}-D_{pre}}{D_{pre}}\times 100\;\%$$ (55)

As shown in Table 5, compared with other approaches, the proposed system has a slight decrease in the average delay time of each intersection for normal traffic, and the delay time of the road network for normal traffic in Low Traffic and Medium Traffic cases. compares the impact of different approaches on general traffic. The proposed system achieves the lowest GTDCR (7.32% to 12.54%) and IQL (14.25–21.73 vehicles) across all traffic conditions, demonstrating its ability to balance EV priority with minimal disruption. In contrast, static methods (e.g., Only signal preemption with A*) exacerbate congestion, with GTDCR exceeding 33.80% and IQL reaching 42.66 vehicles in high traffic. It is worth noting that there is a substantial decline in the two indexes using the proposed system compared with using the other three approaches, since the proposed system could offer lane-level route selection and reduce congestions of road networks, in the High Traffic case. The EVs could maintain efficiency without queuing in all traffic flow cases and cause less delay time for normal traffic when using the proposed system. Further, under higher traffic flow conditions, the times that a signal control strategy is exercised (i.e., 2.9, 7.3, 14.1 in Low Traffic, Medium Traffic, and High Traffic cases, respectively) would be more and the delay time of each intersection and the whole road network are both longer than under lower traffic flow condition, since signal control strategy could influence the behaviors of normal vehicles.

**Table 5:** Average results of impacts brought by EVs on normal traffic.

Case	Approach	Number of times for the EV queuing	Average delay time of each intersection for normal traffic(s)	Delay time of the road network for normal traffic (s)	GTDCR	IQL
Low Traffic	P	**0**	**6.15**	**108.01**	**7.32%**	**14.25**
	O	0	8.23	137.74	12.11%	18.72
	L	0	8.57	140.12	14.55%	20.48
	W	0	8.78	143.67	15.23%	22.18
	S	9.75	------	------	------	------
Medium Traffic	P	**0**	**7.43**	**125.19**	**8.87%**	**16.54**
	O	0	11.09	193.75	19.73%	24.36
	L	0	11.38	198.39	21.55%	26.87
	W	0	11.67	204.94	23.10%	28.95
	S	14.49	------	------	------	------
High Traffic	P	**0**	**11.53**	**198.20**	**12.54%**	**21.73**
	O	0	17.60	309.56	27.63%	35.44
	L	0	18.76	325.37	30.15%	38.98
	W	1.3	20.33	349.48	33.80%	42.66
	S	18.24	------	------	------	------

P: Proposed system; O: Only signal preemption with road-level route planning; L: Lane-level route planning without signal control; W: Only signal preemption with A*; S: Only A*.

**Fig 6:**
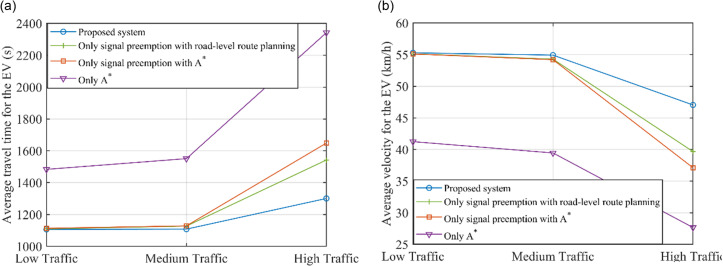
Comparisons of the EVs efficiency under different traffic flow conditions.

Fig 6 shows the average travel time and the average velocity of EV to compare the efficiency of EVs under different traffic flow conditions. Herein, results of lane-level route planning without signal control is not presented since it could bring less travel time to EV but more delay time for normal traffic. As shown in Fig 6, as the traffic flow increases, the average velocity of EVs will decrease and the travel time will increase. However, in all cases, EVs using the proposed system performs better than others. Particularly, the average velocity of EVs is close to the desired speed in low traffic case using the proposed system.

## Discussions

### Compatibility with Partial CV Infrastructure

While CV technology enhances real-time coordination, the proposed system prioritizes compatibility with existing urban sensing infrastructure. For example:

- Loop Detectors: Provide foundational traffic flow data for road-level route planning.
- Mobile Signaling: Compensates for sparse CV data by estimating lane-level vehicle trajectories (Section 3.1).
- Fallback Mechanism: If CV communication is unavailable (e.g., due to device failures), the system dynamically shifts to historical traffic patterns and loop detector inputs.

**Fig 7:**
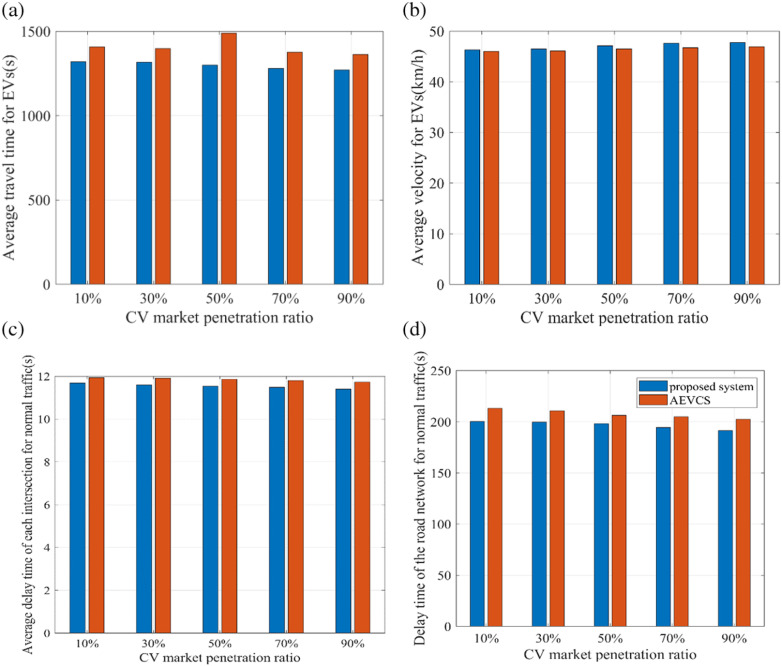
Comparisons of our proposed system and AEVCS under different CV market penetration ratios.

To further verify the effectiveness of the proposed system, five CV market penetration ratios under high traffic cases are examined: 10%, 30%, 50%, 70%, 90%. Likewise, the performance of our proposed system is compared with that of the EV control strategy proposed by [39] labeled as AEVCS, as shown in Fig 7. It can be seen that under high traffic cases, the EVs using our proposed system reach their destination faster and cause less delay time for normal traffic. Moreover, when there exist more CVs, the traffic state estimation results would be more accurate, so that the EVs efficiency can be improved and the adverse impact on normal traffic is reduced.

### Compatibility with communication latency and multi-EVs

**Fig 8:**
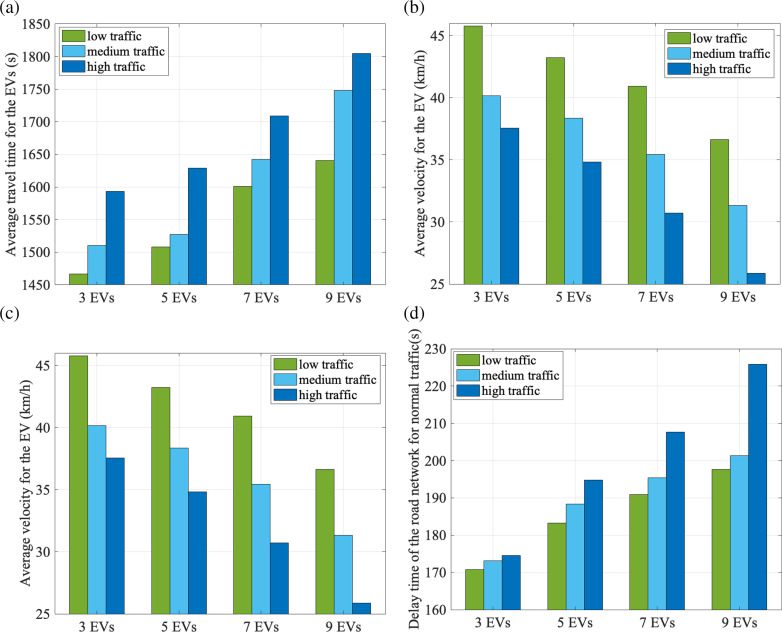
Performance of our proposed system with 0.2-second latency time under different traffic flow conditions.

To evaluate the system’s robustness under realistic communication conditions, we conducted additional experiments introducing 0.2-second latency time respectively in V2I/V2V data transmission as shown in Fig 8. This latency simulates typical delays caused by network congestion or signal processing in 90% CV penetration ratio environments with multi-EVs.

The simulation results indicate that a communication latency of 0.2 seconds introduces moderate performance degradation (8.3% increase in EV travel time and 7.3% rise in general traffic delays). However, these impacts remain within acceptable thresholds, as the system retains its ability to prioritize EVs while mitigating congestion compared to static strategies. Regarding scalability, increasing the number of EVs amplifies their collective impact on normal traffic. Under low-to-medium traffic conditions (e.g., 550–1100 vehicles/hour/lane), the system effectively minimizes disruptions. In high-density scenarios (1600 vehicles/hour/lane), the cumulative effect of multiple EVs raises intersection delays by 6.7% compared to single-EV cases. Nevertheless, the proposed bi-level MPC framework dynamically redistributes traffic pressure through lane-level rerouting and adaptive signal preemption, ensuring that adverse impacts remain manageable. This balance underscores the system’s robustness in heterogeneous environments.

Suppose that the EV platoon is made up of six EVs and the CV market penetration ratios is 50%. As shown in Fig 7, comparing the proposed system with only signal preemption with road-level route planning when the traffic flow is not high, the changes in EV efficiency are not obvious; but in high traffic cases, EV efficiency has been significantly improved. This phenomenon may be due to when providing lane-level route planning, there are fewer vehicles in front of EVs and the road environment is relatively simple. Hence, there is less interference to the speed of EVs in high traffic cases. Then, compared with only signal preemption with A*, EV efficiency is further reduced, owing to dynamic traffic information update and planning, particularly in high traffic cases. Further, without dynamic route planning and signal preemption control, EVs are the least efficient, since they may experience parking and queueing.

In brief, the integrated system of this paper has some advantages. First, it applies rolling horizon to route planning, which can help to adjust the planned route dynamically and improve the efficiency of EVs. Second, the adverse impact of signal preemption on normal traffic is taken into consideration. Through the changes in parameter (i.e., number of nodes for road-level route planning and sampling time) settings, the different situations can be simulated and analyzed. In this case, we examine the case when the number of nodes for road-level route planning and sampling time change.

**Fig 9:**
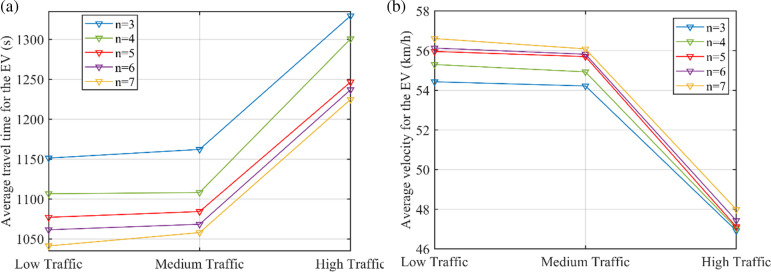
Comparisons of the EVs efficiency under different traffic flow conditions considering the number of nodes selected for road-level route planning.

With the same sampling time, the local road-level planned route might be affected by the number of selected nodes. Fig 9 shows the average travel time and the average velocity for the EVs under different traffic flow conditions considering the number of nodes selected for road-level route planning, with the sampling time of 15s. If fewer nodes are taken into consideration, there is fewer alternative routes for EV to select which has limitations to the choice of road for optimal EV efficiency. Therefore, it takes less traveling time with more selected nodes for EV and the average velocity is promoted, since more route information is given. However, too much route information may slow down the speed of calculation and solution. The number of selected nodes should be set according to the real road environment.

**Fig 10:**
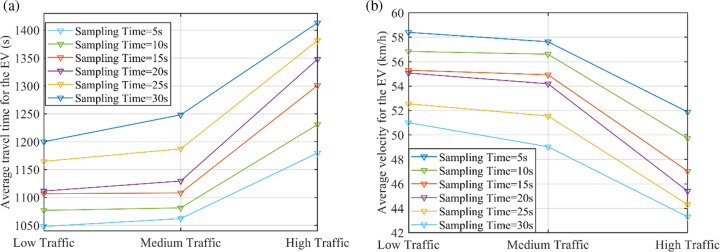
Comparisons of the EV efficiency under different traffic flow conditions considering sampling time.

With the same number of nodes selected, the EVs efficiency could also be affected by sampling time for MPC. Fig 10 shows the average travel time and the average velocity for the EVs under different traffic flow conditions considering sampling time, with four nodes selected. Less sampling time stands for more exquisite perception and optimization. The simulation results show that EV efficiency will increase with less sampling time. However, as a matter of fact, advising an EV on speed and trajectory constantly might bring forced communication abnormalities.

Dynamic route planning and signal preemption control are believed to play a significant role in EVs. On the basis of the simulated results, the integrated system of signal preemption control and route planning can help to maintain EV efficiency and reduce the impact of EVs operation on normal traffic. However, many problems remain to be addressed before wide-scale application is possible—such as the complexity of road network topology building and the high cost of CV constructing. At present, the traffic information can only be constructed using expert data, which is usually acquired by means of multi-layered LiDAR and is very difficult to update online. In future, CV technology will be employed to extract useful information and transfer information.

### Limitations of communication reliability assumptions

The dynamic congestion adjustment enables the system to better handle real-world disruptions. For example, in a simulated accident scenario, the revised model reduced rerouting delays by 15% compared to the original framework. Future work could integrate crowd-sourced incident reports (e.g., via mobile apps) to further improve traffic flow estimation accuracy.

The proposed system assumes reliable communication (i.e., no delays or packet loss) between CVs and infrastructure, with no delays or packet loss. However, in real-world scenarios, communication reliability in partial CV environments may be compromised due to factors such as signal interference, hardware failures, or network congestion. This limitation could affect the accuracy of traffic state estimation and the effectiveness of signal preemption control. For instance, intermittent data loss might lead to outdated traffic flow predictions, delaying dynamic route updates for EVs. Recent studies (e.g., [41] Finkelberg et al., 2022; [42] Petrov et al., 2024) highlight the critical impact of communication reliability on traffic control systems. Finkelberg et al. (2022) demonstrated that even a 10% packet loss rate could degrade intersection control performance by 15%. To address this, future work should integrate robust communication models (e.g., stochastic MPC) or adaptive fault-tolerant mechanisms. Potential solutions include:

- Dynamic Data Fusion: Combining real-time CV data with historical patterns to mitigate sparse data issues.
- Fallback Protocols: Triggering pre-defined emergency routes if communication failures exceed a threshold.
- Edge Computing: Deploying localized decision-making at roadside units to reduce dependency on central servers.

These enhancements would improve the system’s resilience in heterogeneous communication environments.

## Conclusions

In this study, we have proposed an integrated system of signal preemption control and route planning to address the emergency route planning problem for an EV platoon using CV technology in emergency rescue under partial CV environment. A bi-layer MPC optimization framework that dynamically predicts the optimal road and lane for EVs respectively has been developed by traffic flow estimation via data from C-V2X and loop detectors. Further, the signal preemption control scheme combined with lane selecting has been designed to seek an approach to improve the efficiency of EVs and to reduce the impact of EVs operation on normal traffic. Finally, a numerical example and simulations have been provided to illustrate the effectiveness and improvements of the proposed system under different CV market penetration ratios. Potential future work can be focused on (i) how to better utilize vehicle information under C-V2X environment to promote the estimation accuracy of traffic flow characteristics in the road network; (ii) how to take into consideration heterogeneous traffic that consists of automatic vehicles, CVs and traditional human-driven vehicles in the design; (iii) how to better control signal and plan route for EVs if the signal timing of each intersection in the road network is not fixed but changes according to the traffic condition.

## Supporting information

S1 File Adjacency (CSV)

S2 File Support. (CSV)
